# The link between attention deficit hyperactivity disorder (ADHD) symptoms and obesity-related traits: genetic and prenatal explanations

**DOI:** 10.1038/s41398-021-01584-4

**Published:** 2021-09-04

**Authors:** Ville Karhunen, Tom A. Bond, Verena Zuber, Tuula Hurtig, Irma Moilanen, Marjo-Riitta Järvelin, Marina Evangelou, Alina Rodriguez

**Affiliations:** 1 Department of Epidemiology and Biostatistics, Imperial College London, London, UK; 2 The University of Queensland Diamantina Institute, The University of Queensland, Brisbane, Australia; 3 MRC Integrative Epidemiology Unit at the University of Bristol, Bristol, UK; 4 Population Health Sciences, Bristol Medical School, University of Bristol, Bristol, UK; 5 Research Unit of Clinical Neuroscience, Psychiatry, University of Oulu, Oulu, Finland; 6 PEDEGO Research Unit, Child Psychiatry, University of Oulu, Oulu, Finland; 7 Clinic of Child Psychiatry, Oulu University Hospital, Oulu, Finland; 8 Unit of Primary Care, Oulu University Hospital, Oulu, Finland; 9 Center for Life Course Health Research, Faculty of Medicine, University of Oulu, Oulu, Finland; 10 Department of Life Sciences, College of Health and Life Sciences, Brunel University, London, UK; 11 Department of Mathematics, Imperial College London, London, UK; 12 Centre for Psychiatry and Mental Health, Wolfson Institute of Population Health, Queen Mary University London, London, UK

**Keywords:** Clinical genetics, ADHD

## Abstract

Attention-deficit/hyperactivity disorder (ADHD) often co-occurs with obesity, however, the potential causality between the traits remains unclear. We examined both genetic and prenatal evidence for causality using Mendelian Randomisation (MR) and polygenic risk scores (PRS). We conducted bi-directional MR on ADHD liability and six obesity-related traits using summary statistics from the largest available meta-analyses of genome-wide association studies. We also examined the shared genetic aetiology between ADHD symptoms (inattention and hyperactivity) and body mass index (BMI) by PRS association analysis using longitudinal data from Northern Finland Birth Cohort 1986 (NFBC1986, *n* = 2984). Lastly, we examined the impact of the prenatal environment by association analysis of maternal pre-pregnancy BMI and offspring ADHD symptoms, adjusted for PRS of both traits, in NFBC1986 dataset. Through MR analyses, we found evidence for bidirectional causality between ADHD liability and obesity-related traits. PRS association analyses showed evidence for genetic overlap between ADHD symptoms and BMI. We found no evidence for a difference between inattention and hyperactivity symptoms, suggesting that neither symptom subtype is driving the association. We found evidence for association between maternal pre-pregnancy BMI and offspring ADHD symptoms after adjusting for both BMI and ADHD PRS (association *p*-value = 0.027 for inattention, *p* = 0.008 for hyperactivity). These results are consistent with the hypothesis that the co-occurrence between ADHD and obesity has both genetic and prenatal environmental origins.

## Introduction

Attention-deficit/hyperactivity disorder (ADHD) often co-occurs with obesity. There has been a surge of studies investigating this association in recent years, despite some variability in findings, the consensus is that the association is well replicated, yet puzzling [1–4]. Together, the double burden of both conditions accounts for nearly a two-fold increase in the cost of care [5]. Inattention and hyperactivity, the core symptom subtypes of ADHD, interfere with development and functioning [6], even at levels below the clinical threshold [7], and are linked to a negative developmental trajectory [8], whereas obesity is a risk factor for all-cause mortality [9]. Identifying the underlying reasons for this coexistence is crucial for both prevention and treatment. Our goal is to examine the evidence for causality, both genetic and prenatal, that may explain the ADHD-overweight/obesity association using Mendelian Randomisation (MR) and polygenic risk scores (PRS).

**Fig. 1:**
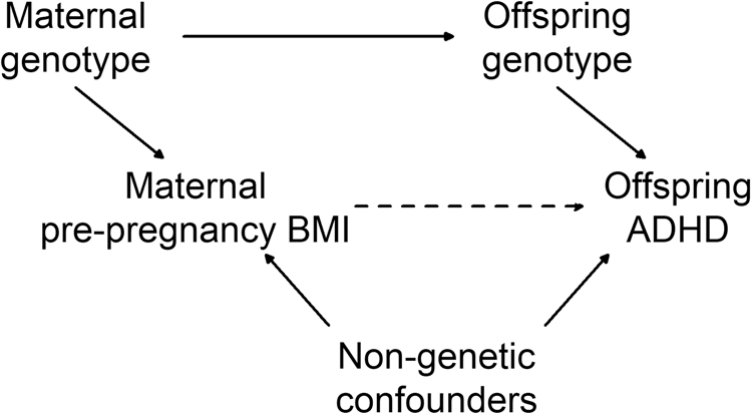
Directed acyclic graph of potential relations between maternal BMI and offspring ADHD. The dashed line represents the effect of interest and solid lines represent other hypothesised associations.

Exposure to maternal obesity at the start of pregnancy was first reported as a risk factor for ADHD symptoms in the child in 2008 [10]. Experimental animal models provide evidence for a causal link between prenatal exposure to maternal obesity and offspring hyperactivity, hypothesized being due to an adverse intrauterine environment [11, 12]. Numerous human studies have replicated the association [13–16], including those assessing endophenotypes of ADHD [17, 18]. However, there is known shared genetic aetiology between ADHD and body mass index (BMI) [19, 20], and genetic familial confounding may account in part for the association between high maternal pre-pregnancy BMI and offspring ADHD [21] as shown in the directed acyclic graph (Fig. 1). This familial co-aggregation, including maternal obesity in pregnancy as a risk factor for child overweight [22], could also account for ADHD and obesity in the child.

MR, a method that uses genetic variants as surrogates for the exposure variable, has the benefit of circumventing issues in observational studies relating to confounding or reverse causation [23]. A few studies have examined the ADHD-obesity link longitudinally in children and reported that the likely direction is from ADHD to obesity, but there is also support for a bidirectional association [3]. Results of bidirectional MR on ADHD and BMI support a causal effect of high BMI on liability to ADHD diagnosis (hereafter ADHD liability), but not vice versa [24]. However, caution should be noted as BMI was the only obesity-related variable used in that study [24]. BMI does not fully reflect body fat or abdominal obesity, the latter being an important risk factor for multiple diseases [25], especially in children [26], thus calling for further investigation.

To address this issue, we investigated the potential causal association between ADHD liability and multiple obesity-related traits in a bidirectional two-sample MR. We undertook various sensitivity analyses relaxing the standard assumptions of MR and used negative control exposure and outcome to validate the specificity of our findings.

To further probe genetic mechanisms, we took advantage of genome-wide association studies (GWAS) on both ADHD liability and BMI. These studies have shown considerable polygenicity [19, 27]. The effects of multiple common DNA variants from GWAS summary statistics can be aggregated to a PRS that summarises individual genetic liability to the phenotype [28]. We utilised PRS for two association analyses. First, we investigated the contribution of inattention and hyperactivity symptoms on the shared genetic aetiology between ADHD and BMI. Secondly, we tested the hypothesis that prenatal exposure to maternal overweight/obesity is associated to ADHD symptoms in childhood independent of the individual genetic liability as measured by PRS.

We used individual-level and longitudinal data from Northern Finland Birth Cohort 1986 (NFBC1986). The use of this pregnancy-offspring cohort provided us with distinctive advantages—we were able to study the shared genetic aetiology of ADHD and adiposity traits at two ages, eight and 16 years, use mother and teacher reports, and had intergenerational data to test the effect of intrauterine exposure to adiposity on offspring ADHD symptoms. In sum, to clarify the causal association between ADHD and obesity, we performed (1) bidirectional MR on ADHD liability and a variety of obesity-related traits, (2) association analysis of both BMI and ADHD PRS with ADHD symptom subtypes, BMI and WHR, and (3) association analysis of maternal pre-pregnancy BMI and offspring ADHD symptoms while adjusting for the inherited genetic susceptibility to the phenotypes.

## Methods

### Data sources

#### GWAS summary statistics

Summary statistics were obtained from the largest and most recent GWAS available for ADHD liability and six obesity-related traits: BMI, waist circumference (WC), waist-hip-ratio (WHR), BMI-adjusted WHR, body fat percentage (BFP) and basal metabolic rate (BMR). Summary statistics for ADHD liability are based on 55 374 individuals (20,183 cases, 35,191 controls) from 12 cohorts [19]. ADHD cases were identified using—depending on the participating study—national registers, interviews or psychiatric diagnoses, detailed in Demontis et al. [19]. Summary statistics for BMI are combined from the GIANT consortium [29] and UKBiobank [30], comprising almost 700,000 individuals [27]. For WHR and BMI-adjusted WHR, we used summary statistics from GIANT consortium [31]. For WC, BFP and BMR, we used summary statistics from UK Biobank provided by the Neale Lab [32]. Table 1 summarises the sources and sample sizes for GWAS of ADHD and obesity-related traits. The original studies had obtained relevant ethical permission and consent.

**Table 1:** Information on GWAS on ADHD and obesity-related traits.

Trait	Abbreviation	Source	*N*
ADHD	ADHD	Demontis et al. [19]	55,374
Body mass index	BMI	Yengo et al. [27]	688 566
Waist circumference	WC	UKBioBank [32]	360 564
Waist-hip-ratio	WHR	GIANT [31]	224 459
BMI-adjusted WHR	WHR (adj. BMI)	GIANT [31]	224 459
Body fat percentage	BFP	UKBiobank [32]	354 628
Basal metabolic rate	BMR	UKBiobank [32]	354 825

#### Participants

NFBC1986 included all pregnant women with expected date of delivery between July 1985 and June 1986 in the two northernmost provinces of Finland [33]. Women were recruited in early pregnancy via antenatal health services; 99% participation was achieved. In total, 9432 children were live-born into the cohort.

Follow-ups have been conducted when children were seven, eight and 16 years old. The Ethical Committee of Northern Ostrobothnia Hospital District approved the study. A written informed consent was provided by parents and adolescents at the 16-year follow-up. Supplementary Fig. 1 shows data collection points and data availability for analysis in this study.

#### ADHD symptoms

Mothers and teachers rated children’s inattention and hyperactivity symptoms at seven and eight years, respectively. Parents used the Rutter A scale to evaluate the children’s behaviour [34], while teachers used the Rutter B2 scale [34]. We constructed inattention and hyperactivity symptom scores separately for each rater based on questions on the core symptoms of inattention and hyperactivity as described earlier in Rodriguez et al. [35] (Supplementary Table 1).

When adolescents were 16 years old, parents used the Strengths and Weaknesses of ADHD symptoms and normal behaviour (SWAN) scale [36]. SWAN measures both weaknesses and strengths and is expected to produce a Gaussian distribution for the inattention and hyperactivity-impulsivity symptoms included in the DSM classification. To make this measurement comparable with the variables at the earlier time point, we used the weakness side of inattention and hyperactivity/impulsivity subscales to generate inattention and hyperactivity/impulsivity symptom scores at 16 years. To ensure the comparison of the scores between raters and time points, all symptom score variables were scaled to range from zero (no symptoms) to two (maximum symptoms).

We also created three global scores to summarise the data by aggregating ADHD symptom scores (i.e., both inattention and hyperactivity/impulsivity symptoms assessed in childhood and in adolescence) across all raters and ages: global inattention, global hyperactivity, and combined global inattention-hyperactivity symptom scores.

#### Anthropometric variables

During the clinical examination at the 16-year follow-up, trained staff measured height, weight, and hip and WCs, from which BMI and WHR were computed. Maternal BMI was extracted from the pregnancy medical record after delivery as previously described [10].

#### Genotype data

Adolescents’ blood samples were obtained during the clinical examination at 16 years. A total of 3834 samples were genotyped using Illumina HumanOmniExpressExome-8v1.2 platform and Beadstudio calling algorithm. After excluding individuals with low call rate (<95%), low mean heterozygosity (<0.305), related individuals (identity-by-descent pairwise sharing <0.2), gender mismatch or duplicate samples, genotype data were available for 3743 adolescents. This includes a selected set of 372 individuals exposed to gestational diabetes, gestational hypertensive disorders and preterm birth, and the rest is a random sample. Adjusting for the sampling stratum in the analyses did not affect the results.

For quality control of the single nucleotide polymorphisms (SNPs), after exclusions based on call rate (<99%) and Hardy-Weinberg equilibrium (HWE, *p* < 1 × 10^−4^), 889,119 SNPs remained in the genotyped dataset. The genetic data were imputed using Haplotype Refererence Consortium (HRC) imputation reference panel. Finally, the imputed dataset was filtered based on imputation quality (excluded SNPs with imputation *R*^*2*^ < 0.5), minor allele frequency (MAF, excluded SNPs with MAF < 0.001) and HWE *p*-value (excluded if *p* < 1 × 10^−12^), and the final genetic data comprised of 11 009 294 SNPs.

Genetic principal components (PCs) were generated using the genotyped SNPs with MAF > 0.05. Prior to the PC calculation, the data were pruned excluding SNPs with *r*^*2*^ > 0.1 to ensure approximate linkage equilibrium.

### Statistical analysis

#### Mendelian randomisation (MR)

We conducted two-sample MR to investigate the potential causality of ADHD liability on obesity-related traits and vice versa. In MR, genetic variants that are robustly associated with the exposure are used as surrogates for the exposure. These variants are then tested for association with the outcome. Both variant-exposure and variant-outcome associations can be extracted from their corresponding GWAS summary statistics [37]. MR analysis can give evidence for a causal effect of the exposure on the outcome, provided that the genetic variants meet instrumental variable assumptions [38]. The inverse variance weighted (IVW) method based on summary data (as proposed by Burgess et al. [37]) was used for the main MR analysis.

Hypothesis testing for the effect of ADHD on multiple obesity-related outcomes requires an appropriate multiple testing correction. We combined the evidence from the IVW MR results by combining the p-values using the harmonic mean *p*-value [39, 40]. This method can be used to combine dependent *p*-values while controlling for family-wise error rate [39].

The IVW method provides an efficient estimate when all of the following instrumental variable assumptions hold: (i) the genetic variants are associated with the exposure, (ii) the genetic variants are independent of all confounders of the exposure-outcome association, and (iii) the genetic variants are independent of the outcome, given the exposure and all confounders. Violations to these assumptions can distort the MR estimates, resulting in inaccurate causal estimates, loss of statistical power or false-positive results [41]. As a sensitivity analysis, we also conducted the MR analysis using weighted median method [42] and MR-PRESSO [43]. These methods are more robust to the violation of assumption (iii), i.e., the genetic variants might be associated with the outcome independently of the exposure, known as horizontal pleiotropy.

Weighted median method provides consistent estimates if at least half of the weight for the analysis comes from valid instrumental variables. MR-PRESSO detects outliers (MR-PRESSO outlier test) in the IVW method and provides outlier-corrected IVW estimates by excluding these outliers from the analysis. MR-PRESSO gives consistent estimates when the assumption for weighted median method and the InSIDE (Instrument Strength Independent of Direct Effect) assumption both hold [43]. We also tested for horizontal pleiotropy using MR-PRESSO Global test [43].

For the MR analyses with ADHD liability as the exposure, we used blonde hair colour as a negative control outcome. This was done to examine the validity of the genetic variants used as instrumental variables for ADHD liability [44]. We would expect no effect from ADHD liability on the negative control outcome. Any detected association would indicate invalidity of these genetic variants as instruments for ADHD liability [44]. We used summary statistics from UK Biobank, provided by the Neale Lab [32].

In addition, to examine the specificity of our findings, we used autism spectrum disorder (ASD), representing a neurodevelopmental disorder, and rheumatoid arthritis (RA), representing an autoimmune disease, as negative control exposures for MR analysis of the effect of ADHD liability to obesity-related traits. These phenotypes were selected as they are based on GWAS of similar size as for ADHD [45, 46].

#### Selection of genetic variants for MR

SNPs that were strongly associated with the exposure (*p* < 1 × 10^−8^ for BMI as suggested in [27], *p* < 5 × 10^−8^ for other obesity-related traits and ADHD liability) were selected as surrogates for the exposure. The effect estimates of these SNPs were extracted from the GWAS summary statistics of both the exposure and the outcome and aligned to have the same effect allele. The SNPs were clumped [47] using a window of 1000 kb and an *r*^2^ threshold of 0.01. For those SNPs not available in the outcome summary statistics, proxies were sought using an *r*^2^ cut-off of 0.8. We tested alternative values for *p*-value threshold (*p* < 1 × 10^−7^ for ADHD liability), clumping window (500 kb), clumping *r*^*2*^ (0.001, 0.1) and proxy *r*^*2*^ cut-off (1.0).

#### Polygenic risk scores (PRS)

We generated PRS for BMI and ADHD. The effect size estimates for each SNP were obtained from the corresponding GWAS summary statistics (Table 1) as the weights. The number of SNPs *M* was optimised by first clumping [47] the summary statistics (clumping window of 250 kb and *r*^*2*^ = 0.01) and then calculating the PRS with *p*-value thresholds of 1 × 10^−8^, 5 × 10^−8^, 1 × 10^−5^, 1 × 10^−4^, 0.001, 0.01, 0.1, 0.2, 0.3, 0.4, and 0.5 using PRSice software [28]. Clinically measured BMI at 16 years and global ADHD symptom score were used as the target phenotypes for BMI and ADHD PRS, respectively. The PRS with the highest *R*^*2*^ with the target phenotype was selected as the final PRS for the corresponding trait.

### PRS association analysis

We conducted association analysis using both ADHD and BMI PRS in NFBC1986 to study the shared genetic aetiology between BMI and ADHD symptom subtypes. Multiple outcomes ordinal regression with logit link function was used to estimate the effect of BMI PRS on the different ADHD symptom outcomes. Ordinal regression is a semiparametric regression approach that requires the outcome to be ordinal or continuous. This method has less restrictive assumptions than the ordinary least squares regression and is invariant to the choice of transformation of the outcome [48]. Using a model for multiple outcomes allows a direct comparison of the effect size estimates of BMI PRS on inattention and hyperactivity symptoms while taking their mutual correlation into account [49]. We report the effect estimates of BMI PRS on the multivariate outcomes adjusted for sex and the first ten genetic PCs. Similarly, we studied the effect of ADHD PRS on adolescent BMI and WHR measured during the clinical examination, using linear regression and the same adjustments as above.

### Maternal pre-pregnancy BMI and offspring ADHD symptoms

We studied our previously reported association between maternal pre-pregnancy BMI and offspring ADHD symptoms as reported by teachers [10], adjusted for both ADHD PRS and BMI PRS. Ordinal regression with a logit link function was used for the analysis. Maternal pre-pregnancy BMI was used as a continuous variable, calculated based on self-reported height and weight before pregnancy at the first antenatal healthcare visit (approximately gestational week 10) and recorded by the attending midwife. Parity, maternal education, smoking during pregnancy, age at delivery and offspring sex were included as additional explanatory variables in the model to adjust for potential confounding. As previous literature shows a J-shaped association between maternal BMI and offspring ADHD risk [14], restricted cubic splines with three knots were used to allow non-linear effects for maternal BMI and other continuous explanatory variables. The added value of adding PRS into models was measured by the relative change in *R*^*2*^ [48].

## Results

### Mendelian Randomisation

**Fig. 2:**
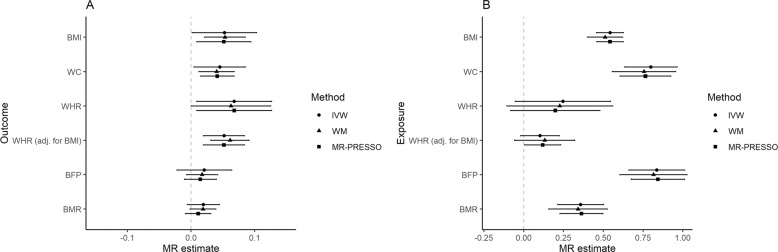
Forest plot of MR estimates and their 95% confidence intervals for the effect of ADHD liability on obesity-related traits (left panel), and for the effect of obesity-related traits on ADHD (right panel). BMI body mass index, WC waist circumference, WHR waist-hip-ratio, BFP body fat percentage, BMR basal metabolic rate, IVW inverse-variance weighted estimate, WM weighted median estimate.

Depending on the trait, the number of independent SNPs available for MR with ADHD liability as the exposure was between nine and 12 (Supplementary Table 2). The MR results of the effect of ADHD liability on obesity-related traits are summarised in Fig. 2 (left panel). The main MR analysis using IVW method showed evidence for ADHD liability-related genetic variants being associated with higher BMI (MR point estimate 0.053, 95% confidence interval [CI, 0.002, 0.103], *p*-value = 0.04), WC (0.045 [0.005, 0.086], *p* = 0.03), WHR (0.068 [0.009, 0.127], *p* = 0.03) and BMI-adjusted WHR (0.052 [0.020, 0.084], *p* = 0.006). The point estimates for the other adiposity traits were also positive (BFP: 0.021 [−0.022, 0.064], *p* = 0.31; BMR: 0.019 [−0.001, 0.045], *p* = 0.12).

Combining the evidence from all MR results using IVW method yielded a harmonic *p*-value of 0.025. Using different parameters for clumping window, clumping *r*^*2*^ or proxy *r*^*2*^ threshold as described in the methods did not have a notable effect on the MR estimates (Supplementary Table 3).

Similar results were observed using weighted median and MR-PRESSO methods, which are more robust to horizontal pleiotropy (Fig. 2). All effect size estimates of genetically determined ADHD liability on obesity-related traits using weighted median method were similar to the IVW estimates. MR-PRESSO Global test detected evidence for horizontal pleiotropy for all outcomes except WHR. Removing the potentially pleiotropic SNPs did not notably affect the effect size estimates, as demonstrated by the similarity of IVW and MR-PRESSO estimates (Fig. 2).

In the negative control outcome analysis, we found no evidence for association between genetically determined ADHD liability and hair colour (IVW estimate 0.002 [−0.010, 0.014], *p* = 0.78). For negative control exposure analyses, we did not find evidence for genetically determined ASD or RA being associated with obesity-related traits (Supplementary Table 4).

In MR analyses treating obesity-related traits as exposures and ADHD liability as the outcome, genetically determined BMI, WC, BFP and BMR were associated with higher ADHD liability (Fig. 2, right panel). Positive effect size estimates were detected for WHR and BMI-adjusted WHR. The results from sensitivity analyses accounting for horizontal pleiotropy had similar effect sizes as the IVW method (Fig. 2).

### PRS association analysis

Descriptive statistics for the observational data are in Supplementary Table 5 and the histogram for ADHD global symptom score in Supplementary Fig. 2. The *R*^*2*^ values for the PRS and outcomes are reported in Supplementary Table 6.

**Fig. 3:**
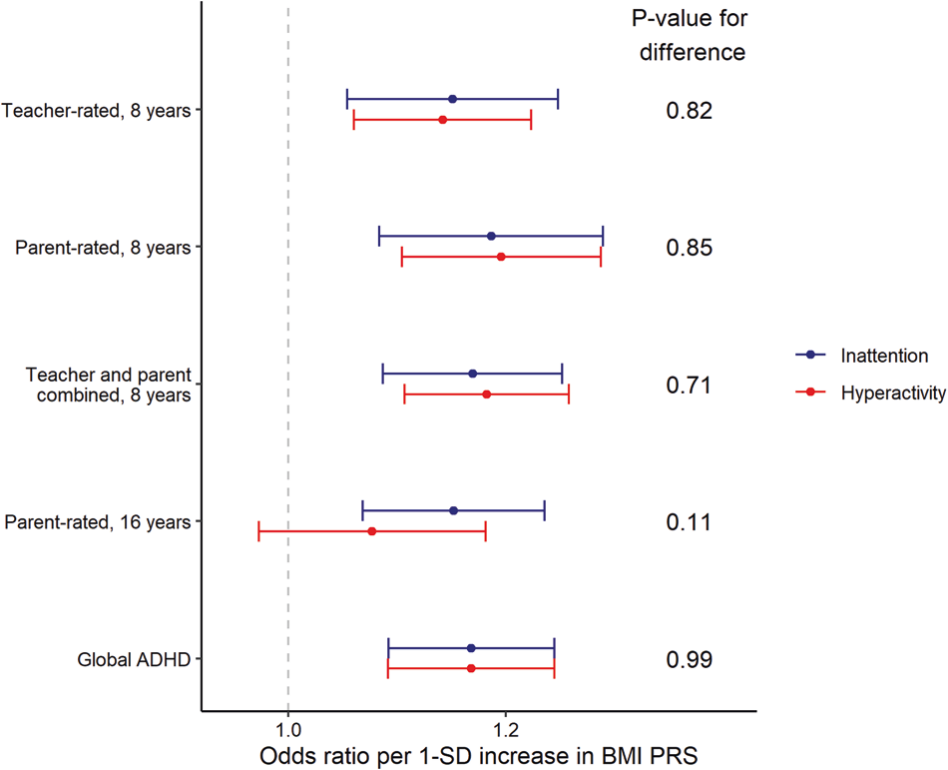
Effect size estimates and their 95% confidence intervals per 1-standard deviation increase in BMI PRS on increasing number of ADHD symptom types according to rater and age. *P* values are for testing the difference between inattention and hyperactivity effect size estimates.

We found BMI PRS being associated with both global inattention and global hyperactivity. The odds ratios (OR) and their CIs for increasing number of symptoms per 1-standard-deviation (SD) increase in BMI PRS were identical for both inattention and hyperactivity (OR = 1.17, 95% CI [1.09, 1.25], *p*-value for difference of estimates = 0.99). Analysing each rater and time point separately yielded similar effect size estimates (Fig. 3). BMI PRS was associated with global inattention-hyperactivity score (OR per 1-SD increase in BMI PRS 1.17, 95% CI [1.10, 1.24]), and ADHD PRS was associated with BMI (SD-change per 1-SD increase in ADHD PRS 0.07, 95% CI [0.04, 0.11]). The effect size point estimate for the association between ADHD PRS and WHR was also positive (SD-change per 1-SD increase in ADHD PRS 0.02, 95% CI [−0.01, 0.05]).

### Maternal pre-pregnancy BMI and offspring ADHD symptoms

**Fig. 4:**
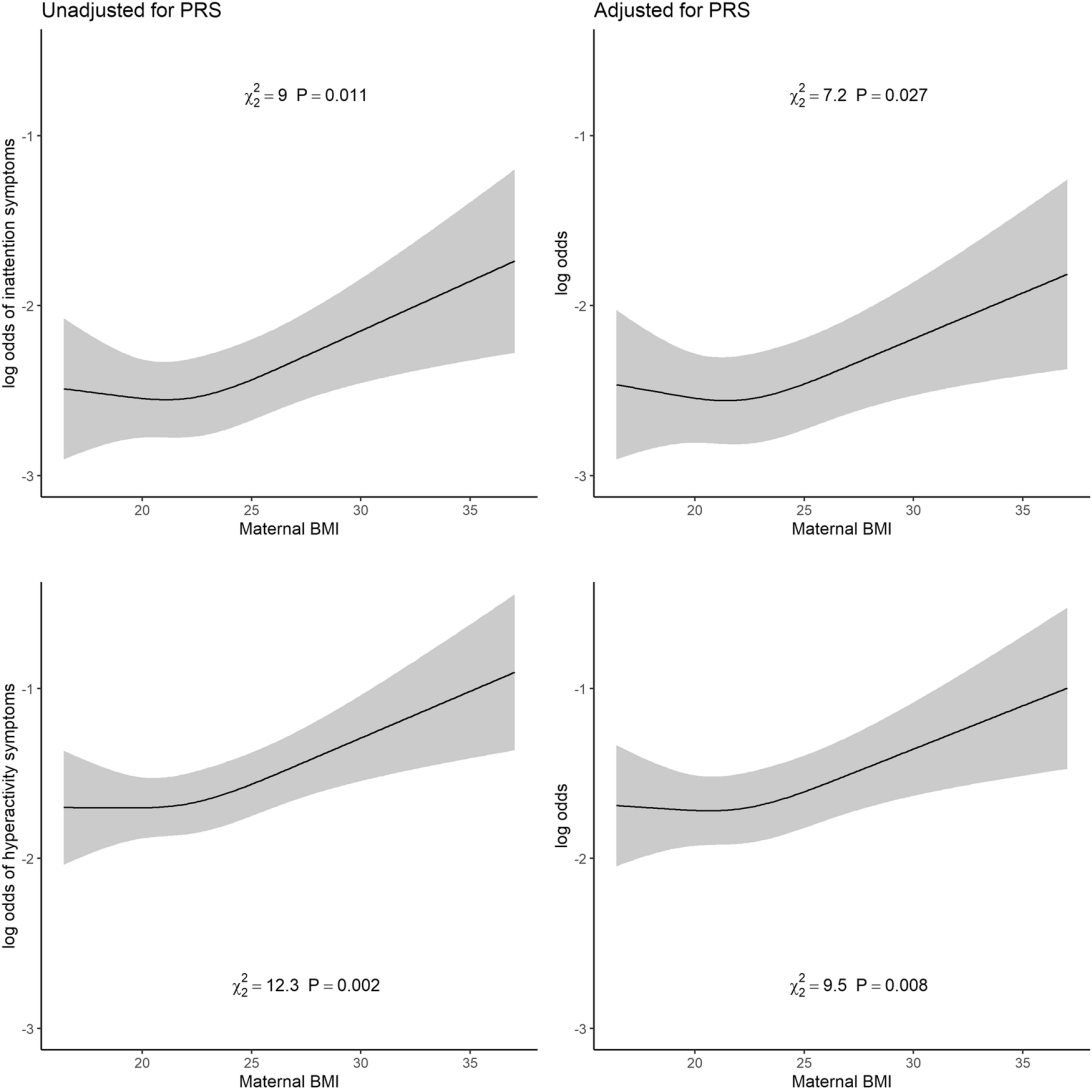
Association between maternal pre-pregnancy BMI and the log-odds of increasing number of teacher-rated inattention (top panels) and hyperactivity (bottom panels) symptoms at eight years, adjusted for parity, maternal education, smoking during pregnancy, age at delivery and offspring sex. Left panels show the associations without an additional adjustment for PRS of ADHD and BMI, and right panels show the associations with the adjustment.

Maternal pre-pregnancy BMI was associated with offspring ADHD symptoms rated by teachers after adjusting for both BMI and ADHD PRS. The associations before and after adjustment for offspring BMI PRS and ADHD PRS are characterised in Fig. 4. The *p*-values for testing the null hypothesis of no association in the PRS-adjusted model were 0.027 and 0.008 for inattention and hyperactivity, respectively. The relative change in *R*^*2*^ when PRS were included in the model was 0.030 for inattention and 0.026 for hyperactivity.

## Discussion

Our results support the hypothesis that the co-occurrence between ADHD and obesity has both genetic and prenatal origins. First, we conducted a bidirectional MR on ADHD liability and five obesity-related traits. We found consistent evidence for a bidirectional causal association. Second, using individual-level data, we studied the genetic architecture and found the genetic liability to BMI being associated with both inattention and hyperactivity symptoms in childhood and adolescence. Third, using prospective longitudinal data, we found evidence that prenatal exposure to maternal overweight/obesity was associated with an increased risk of ADHD symptoms in children after accounting for the genetic risk and a host of confounders.

Our results bolster the suggested bidirectional association derived from evidence across observational studies [3]. Different mechanisms are likely to underlie each causal direction; ADHD may lead to higher obesity risk possibly due to abnormal functioning of the dopamine pathway that leads to decreased physical activity and increased sedentary lifestyle [4]. Recent evidence shows that physical activity is associated with brain development and attention networks thought to be involved in ADHD [50]. Further, obesity is known to trigger systemic chronic inflammation [51], which is consequently suggested to have an impact on brain functions, including ADHD-type symptoms [4, 52].

Our results partially contrast with the only other MR analysis of ADHD liability and obesity [24], which reported no consistent evidence for the ADHD liability to BMI pathway. In contrast, our study was more comprehensive because we used six traits to measure obesity, rather than relying solely on BMI. We used weighted median and MR-PRESSO methods as sensitivity analyses to examine the effect of possible violations to MR assumptions and found the results to be robust throughout. We also used negative controls to assess the validity of the genetic instrument for ADHD liability and specificity of the findings. There was no evidence for association between genetically predicted ADHD liability and hair colour, i.e., the negative control outcome, and no evidence for association between ASD or RA (i.e. negative control exposures) and BMI. Together these results provide confidence that the bidirectional ADHD-obesity effects are not spurious.

We studied the genetic overlap between inattention, hyperactivity and the combined symptoms with BMI using PRS. PRS offers the advantage of summarising genetic effects and providing a polygenic signal from a set of markers that individually may explain only a small fraction of the trait’s variance. In this way, we replicated the earlier evidence for the genetic overlap between BMI and ADHD [20]. We add to this evidence by assessing symptoms separately and globally both in childhood and adolescence, using two informants (teachers and parents), which indicates that the results are robust across ages and raters in our sample of 2984 participants. We found no evidence for differences in the associations between BMI PRS and inattention and hyperactivity symptoms, suggesting that neither type of symptom is driving the shared genetic aetiology with BMI. We also found evidence that ADHD PRS was associated with clinically measured BMI in the same sample. We report a positive association with ADHD PRS and WHR, although the CIs overlapped with zero. This is likely due to the relatively weak power of ADHD PRS, as the power of a PRS is dependent on the GWAS sample size [53].

We tested our original finding from a cross-country sample, including over 13,000 teacher reports, showing that maternal pre-pregnancy overweight/obesity predicted ADHD symptoms in children [10] by re-testing the NFBC1986 sub-sample. Here, we included PRS for BMI and ADHD as well as various confounders as covariates in our model. To our knowledge, this is the first study examining prenatal exposure to maternal overweight/obesity and child ADHD symptoms while taking direct genetic impact into account. Our present results provide evidence for an independent association and underline the importance of the prenatal environment as a key determinant of child neurodevelopment. A proof-of-concept study in mice experimentally demonstrated that maternal obesogenic diet during pregnancy altered offspring corticogenesis and manifested as anxiety-like behaviours [54]. One potential mechanism would be via adipokines related to maternal obesity which can impact placental function by upregulating the serotonergic system and in turn over-exposes the fetus resulting in a lessening of axonal growth [55–57].

Our results should be interpreted in light of the study’s limitations. One limitation for MR is the interpretation of the MR effect size estimate with a binary exposure. We obtained the genetic variants associated with ADHD from a GWAS where ADHD was considered as a binary trait, representing only the extreme of a continuous dimension of ADHD symptoms. When using a binary exposure, the MR point estimate does not have a clear interpretation [58]. However, testing for the causal null hypothesis is still valid [41]. In our results, we concentrate on the hypothesis testing, and the point estimates and their confidence intervals are reported for the sake of completeness.

We used the GWAS summary statistics that are currently available which means that the genetic information on ADHD liability is based on diagnosed ADHD patients and includes just over 55,000 individuals in total (cases and controls), thus the precision for ADHD MR and PRS contrasts with the GWAS data for the adiposity traits; our BMI PRS was based on summary statistics of almost 700,000 people. Despite this difference, our negative control analyses showed that the genetic instruments functioned as we aimed. As of writing, no summary statistics from substantially large GWAS are available for inattention and hyperactivity, and thus, sufficiently powered MR analysis or PRS for the separate ADHD symptom sub-types are not yet feasible. Nonetheless, we are first in examining inattention and hyperactivity symptom sub-types, which are the core of ADHD, using different raters and in childhood and adolescence.

We had data available on all 18 symptoms of ADHD only at the 16-year report via the SWAN. Consequently, the global ADHD symptom score we used includes impulsivity symptoms at 16 years but not in childhood, thus somewhat limiting conclusions in regard to impulsivity. A recent study also using PRS for ADHD and BMI, based on <900 participants, proposed neural substrates related to impulsivity could be the link between the two conditions, i.e., via unmeasured impulsive eating [59]. However, there are conflicting reports concerning the dietary and physical activity habits of children with ADHD, some reporting no differences [60] while others do [61]. Thus, the study of behavioural impulsivity and its genetic architecture in relation to obesity merits further study.

For the association between maternal pre-pregnancy BMI and offspring ADHD symptoms, we were only able to use the genotype of the offspring for the adjustment. As the offspring genotype is also influenced by father’s genotype, adjusting for the offspring genotype might cause bias in the results due to the fact that father’s genotype may affect offspring ADHD symptoms via other pathways than through offspring PRS only. In addition, information on parents’ ADHD symptoms was not available to use as covariates. While the use of offspring PRS partially captures the genetic effects, parental ADHD symptoms may exacerbate child ADHD via alternative pathways, such as parenting style [62]. A more stringent test of the prenatal environment would include genetic data from the family trio as well as reports of ADHD symptoms, because this is seldom available our analyses included only the BMI and ADHD PRSs for the offspring, notwithstanding this represents the individual’s genetic risk.

In the individual participant data analysis using NFBC1986, our approach was to study symptoms of ADHD rather than diagnosis. We do not view this necessarily as a limitation, but rather as a strength. ADHD is the extreme of a continuous trait, which, even at sub-threshold level, is linked to impairments [7, 35]. Likewise, we examine BMI and WHR as a continuous traits. Thus, our results apply for the full spectrum of the population rather than extreme or diagnosed cases, which is subject to biases, including healthcare accessibility. We measured ADHD symptoms longitudinally in the same individuals in childhood and adolescence. Our findings support the inference that the genetic impact is comparable across development. Further, we used maternal and teacher ratings of symptoms and found that the results were similar across rater and in our composite global measure, suggesting that our data were not affected by rater bias. An important feature that adds to the precision and sensitivity of our results is that participants were all the same age at the times of assessment. Our sample was between 7 and 8 years old in childhood or 16 in adolescence. Although symptoms are known to vary across ages, many studies include a wide range of ages, which can add heterogeneity to the phenotype.

Another common source of variability is self-reported BMI. However, here we used clinically measured offspring height, weight as well as waist and hip circumferences and maternal BMI abstracted from medical prenatal records, thus increasing the accuracy of measurement. Importantly, we examine the impact of the prenatal environment while adjusting for the genetic risk.

Within the bounds of these strengths and limitations, given that our data sources are based on observational studies, the implication of the present work is that there are genetic and prenatal components explaining the ADHD-obesity association repeatedly reported in observational studies. Therefore, these results create opportunities for treatment and prevention. The risk of obesity needs to be considered when treating ADHD symptoms and ADHD screening is advisable for children and adolescents seeking care for obesity, due to the shared genetic liability. It is likely that in humans there is continuity between the obesogenic environment during prenatal development and childhood/adolescence, thus genetic and environmental factors are likely to have an additive effect. In turn, obese adolescents, bearing genetic risk for ADHD, are likely to enter pregnancy overweight/obese thus compounding the risk of ADHD symptoms in their child and contributing to a vicious cycle. Our results reinforce the importance of maternal overweight/obesity prior to pregnancy as a time for the promotion of health not only for an immediate, but for a trans-generational benefit.

## Supplementary information

The link between Attention Deficit Hyperactivity Disorder (ADHD) symptoms and obesity-related traits: Genetic and prenatal explanations Supplementary information
