# Supplementary material for: The link between attention deficit hyperactivity disorder (ADHD) symptoms and obesity-related traits: genetic and prenatal explanations
Source: Transl Psychiatry. 2021 Sep 4;11:455. doi: 10.1038/s41398-021-01584-4 (PMC8418601; doi:10.1038/s41398-021-01584-4)
Supplement: Supplementary file 1 — The link between Attention Deficit Hyperactivity Disorder (ADHD) symptoms and obesity-related traits: Genetic and prenatal explanations Supplementary information [file 41398_2021_1584_MOESM1_ESM.docx]

The link between Attention Deficit Hyperactivity Disorder (ADHD) symptoms and obesity-related traits: Genetic and prenatal explanations

Supplementary information

This supplementary information contains Supplementary Figures 1-2 (pages 2-3) and Supplementary Tables 1-6 (pages 4-9).

**
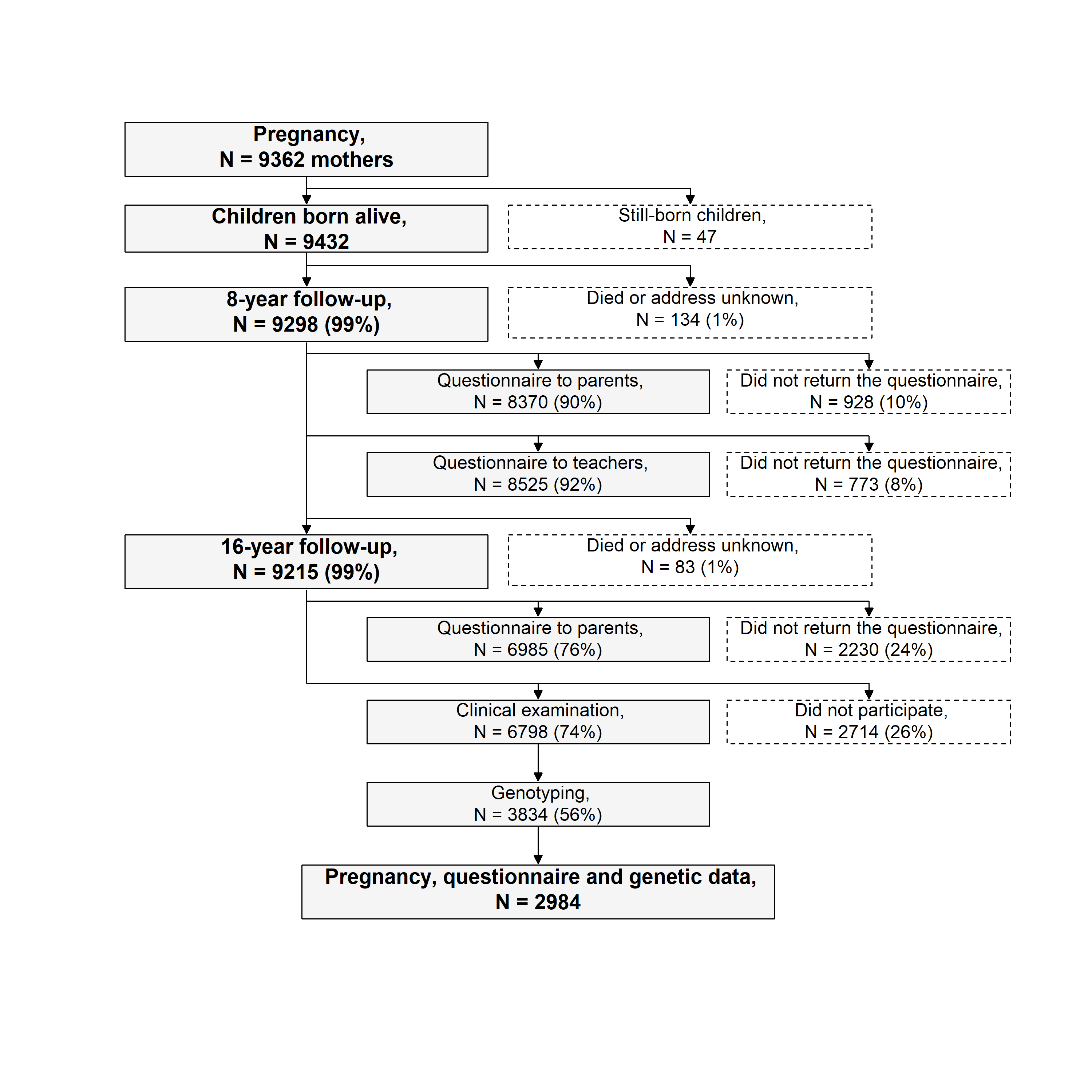
**

**Supplementary Figure 1.** Flowchart of the data collections in the Northern Finland Birth Cohort 1986 used in this study.

**
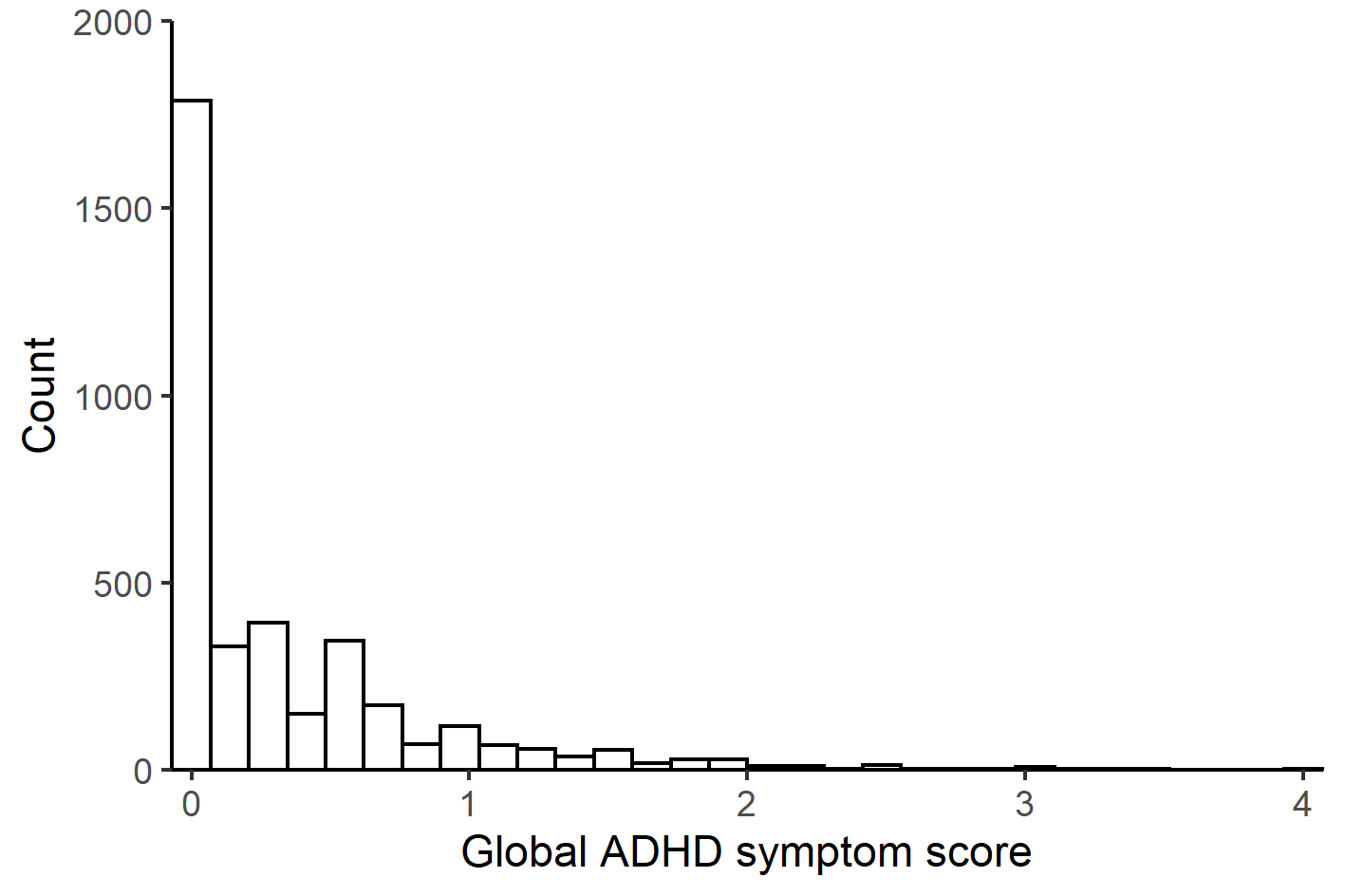
**

**Supplementary Figure 2.** Histogram for the global ADHD symptom score in Northern Finland Birth Cohort 1986, N = 2984.

| Age | Rater | Symptom | Question(s) | Scale |
| --- | --- | --- | --- | --- |
| 8 | Parent | Inattention | Rutter A scale: He/she is not able to concentrate on anything for a longer period of time | 0 = does not fit; 1 = fits partly; 2 = fits well |
| 8 | Parent | Hyperactivity | Rutter A scale: He/she is very restless and it is hard for him/her to sit down | 0 = does not fit; 1 = fits partly; 2 = fits well |
| 8 | Teacher | Inattention | Rutter B2 scale: He/she is not able to concentrate on anything for a longish period of time | 0 = does not fit; 1 = fits partly; 2 = fits well |
| 8 | Teacher | Hyperactivity | Rutter B2 scale: Child wriggles and is restless  Child is restless, does not have patience to sit down for a longish period of time | 0 = does not fit; 1 = fits partly; 2 = fits well  0 = does not fit; 1 = fits partly; 2 = fits well |
| 16 | Parent | Inattention | SWAN rating scale | Scaled score, 0 = no symptoms; 2 = maximum symptoms |
| 16 | Parent | Hyperactivity | SWAN rating scale | Scaled score, 0 = no symptoms; 2 = maximum symptoms |

**Supplementary Table 1.** Questions assessing the core symptoms of attention-deficit hyperactivity disorder by each rater at each time point. The global inattention, global hyperactivity and combined global inattention-hyperactivity symptom scores were obtained by aggregating the symptom scores across all ages and raters for inattention, hyperactivity and all items, respectively. SWAN: Strengths and Weaknesses of ADHD symptoms and Normal behaviour.

|  | BMI | WC | WHR | WHR (adj. BMI) | BFP | BMR |
| --- | --- | --- | --- | --- | --- | --- |
| rs11420276 | rs12410155 | Y | rs12410155 | rs12410155 | Y | Y |
| rs1222063 | rs2391769 | Y | rs2391769 | rs2391769 | Y | Y |
| rs9677504 | Y* | Y | Y | Y | Y | Y |
| rs4858241 | Y | Y |  |  | Y* | Y |
| rs28411770 |  | Y* |  |  | Y* | Y |
| rs4916723 | Y | Y | Y | Y | Y | Y |
| rs5886709 | rs10262192 | rs10262192 | Y | Y | rs10262192 | rs10262192 |
| rs74760947 |  | Y |  |  | Y | Y |
| rs11591402 | Y | Y | Y | Y | Y | Y |
| rs1427829 | Y* | Y* | Y | Y | Y* | Y* |
| rs281324 | rs281320* | Y | rs281320 | rs281320 | Y* | Y |
| rs212178 | rs12596294* | Y | rs12596294 | rs12596294 | Y | Y |

**Supplementary Table 2.** Genetic variants used in Mendelian Randomisation analyses with ADHD as exposure and obesity-related traits as outcomes. If a proxy SNP was used, this SNP is given. Asterisk denotes that the SNP was removed from MR-PRESSO analysis due to evidence for heterogeneity.

| P-value threshold for instruments | Clumping parameters | |  |  |  |  |
| --- | --- | --- | --- | --- | --- | --- |
|  | window | r^2^ | Proxy r^2^ | SNPs | B (CI) | p |
| 1e-07 | 500 | 0.01 | 0.8 | 12 | 0.060 (0.014; 0.105) | 0.015 |
|  |  |  | 1.0 | 12 | 0.060 (0.014; 0.105) | 0.015 |
|  |  | 0.10 | 0.8 | 16 | 0.058 (0.017; 0.099) | 0.009 |
|  |  |  | 1.0 | 16 | 0.058 (0.017; 0.099) | 0.009 |
|  | 1000 | 0.01 | 0.8 | 12 | 0.060 (0.014; 0.105) | 0.015 |
|  |  |  | 1.0 | 12 | 0.060 (0.014; 0.105) | 0.015 |
|  |  | 0.10 | 0.8 | 16 | 0.058 (0.017; 0.099) | 0.009 |
|  |  |  | 1.0 | 16 | 0.058 (0.017; 0.099) | 0.009 |
|  | 10000 | 0.01 | 0.8 | 12 | 0.060 (0.014; 0.105) | 0.015 |
|  |  |  | 1.0 | 12 | 0.060 (0.014; 0.105) | 0.015 |
|  |  | 0.10 | 0.8 | 16 | 0.058 (0.017; 0.099) | 0.009 |
|  |  |  | 1.0 | 16 | 0.058 (0.017; 0.099) | 0.009 |
| 5e-8 | 500 | 0.01 | 0.8 | 10 | 0.053 (0.002; 0.103) | 0.043 |
|  |  |  | 1.0 | 10 | 0.053 (0.002; 0.103) | 0.043 |
|  |  | 0.10 | 0.8 | 12 | 0.041 (-0.007; 0.089) | 0.086 |
|  |  |  | 1.0 | 12 | 0.041 (-0.007; 0.089) | 0.086 |
|  | 1000 | 0.01 | 0.8 | 10 | 0.053 (0.002; 0.103) | 0.043 |
|  |  |  | 1.0 | 10 | 0.053 (0.002; 0.103) | 0.043 |
|  |  | 0.10 | 0.8 | 12 | 0.041 (-0.007; 0.089) | 0.086 |
|  |  |  | 1.0 | 12 | 0.041 (-0.007; 0.089) | 0.086 |
|  | 10000 | 0.01 | 0.8 | 10 | 0.053 (0.002; 0.103) | 0.043 |
|  |  |  | 1.0 | 10 | 0.053 (0.002; 0.103) | 0.043 |
|  |  | 0.10 | 0.8 | 12 | 0.041 (-0.007; 0.089) | 0.086 |
|  |  |  | 1.0 | 12 | 0.041 (-0.007; 0.089) | 0.086 |

**Supplementary Table 3.** Mendelian Randomisation results for the effect of ADHD on BMI using different parameters for selecting variants.

| Negative control | | Method | SNPs | Beta (95% CI) | P-value | P-value for pleiotropy |
| --- | --- | --- | --- | --- | --- | --- |
| Outcome | Hair colour | IVW | 12 | 0.002 (-0.010; 0.014) | 0.778 |  |
|  |  | WM | 12 | 0.007 (-0.002; 0.016) | 0.106 |  |
|  |  | MR-PRESSO | 11 | 0.006 (-0.000; 0.013) | 0.058 | <0.001 |
| Exposure | ASD | IVW | 7 | -0.045 (-0.192; 0.102) | 0.483 |  |
|  |  | WM | 7 | 0.006 (-0.039; 0.050) | 0.760 |  |
|  |  | MR-PRESSO | 2 | 0.004 (-0.108; 0.115) | 0.756 | <0.001 |
|  | RA | IVW | 46 | -0.006 (-0.013; 0.001) | 0.115 |  |
|  |  | WM | 46 | -0.002 (-0.006; 0.003) | 0.429 |  |
|  |  | MR-PRESSO | 36 | -0.005 (-0.009; -0.000) | 0.049 | <0.001 |

**Supplementary Table 4.** Mendelian Randomisation results for the effect of ADHD on negative control outcome and negative control exposures with different methods.

|  |  | N (%) or Median (IQR) |
| --- | --- | --- |
| Sex | Male | 1448 (49) |
|  | Female | 1536 (51) |
| Maternal smoking | No | 2381 (80) |
|  | Yes | 603 (20) |
| Maternal education | Basic | 755 (25) |
|  | Upper secondary | 1982 (66) |
|  | Tertiary | 247 (8) |
| Maternal BMI |  | 21.7 (20.1 - 23.8) |
| Parity |  | 1 (0 - 2) |
| Mother’s age at delivery |  | 27.0 (24.0 - 31.0) |
| Parent-rated symptoms at 8 years | Inattention | 0 (0 - 0) |
|  | Hyperactivity | 0 (0 - 0) |
| Teacher-rated symptoms at 8 years | Inattention | 0 (0 - 0) |
|  | Hyperactivity | 0 (0 - 0.5) |
| Combined symptoms at 8 years | Inattention | 0 (0 - 0.5) |
|  | Hyperactivity | 0 (0 - 0.5) |
| Parent-rated symptoms at 16 years | Inattention | 0 (0 - 0) |
|  | Hyperactivity | 0 (0 - 0.1) |
| Global inattention score |  | 0 (0 - 0.2) |
| Global hyperactivity score |  | 0 (0 - 0.2) |
| Combined global score |  | 0 (0 - 0.5) |

**Supplementary Table 5.** Descriptive statistics for the observational data in NFBC1986.

| PRS | outcome | $R^{2}$ |
| --- | --- | --- |
| BMI | BMI | 0.088 |
|  | Global ADHD symptom score | 0.007 |
| ADHD | BMI | 0.005 |
|  | Global ADHD symptom score | 0.008 |

**Supplementary Table 6.** $R^{2}$ values for the association between polygenic risk scores (PRS) and outcomes in Northern Finland Birth Cohort 1986.
